# KL-6 as an Immunological Biomarker Predicts the Severity, Progression, Acute Exacerbation, and Poor Outcomes of Interstitial Lung Disease: A Systematic Review and Meta-Analysis

**DOI:** 10.3389/fimmu.2021.745233

**Published:** 2021-12-09

**Authors:** Tao Zhang, Ping Shen, Chunyan Duan, Lingyun Gao

**Affiliations:** ^1^ School of Medicine, Zunyi Medical University, Zunyi, China; ^2^ Sichuan Academy of Medical Sciences, Sichuan Provincial People’s Hospital, Chengdu, China; ^3^ Medical College, University of Electronic Science and Technology of China, Chengdu, China

**Keywords:** interstitial lung disease (ILD), Krebs von den Lungen-6 (KL-6), progression, severity, acute exacerbation (AE), inflammatory

## Abstract

**Object:**

Interstitial lung disease (ILD) is a specific form of chronic fibrosing interstitial pneumonia with various etiology. The severity and progression of ILD usually predict the poor outcomes of ILD. Otherwise, Krebs von den Lungen-6 (KL-6) is a potential immunological biomarker reflecting the severity and progression of ILD. This meta-analysis is to clarify the predictive value of elevated KL-6 levels in ILD.

**Method:**

EBSCO, PubMed, and Cochrane were systematically searched for articles exploring the prognosis of ILD published between January 1980 and April 2021. The Weighted Mean Difference (WMD) and 95% Confidence Interval (CI) were computed as the effect sizes for comparisons between groups. For the relationship between adverse outcome and elevated KL-6 concentration, Hazard Ratio (HR), and its 95%CI were used to estimate the risk factor of ILD.

**Result:**

Our result showed that ILD patients in severe and progressive groups had higher KL-6 levels, and the KL-6 level of patients in the severe ILD was 703.41 (U/ml) than in mild ILD. The KL-6 level in progressive ILD group was 325.98 (U/ml) higher than that in the non-progressive ILD group. Secondly, the KL-6 level of patients in acute exacerbation (AE) of ILD was 545.44 (U/ml) higher than stable ILD. Lastly, the higher KL-6 level in ILD patients predicted poor outcomes. The KL-6 level in death of ILD was 383.53 (U/ml) higher than in survivors of ILD. The pooled HR (95%CI) about elevated KL-6 level predicting the mortality of ILD was 2.05 (1.50–2.78), and the HR (95%CI) for progression of ILD was 1.98 (1.07–3.67).

**Conclusion:**

The elevated KL-6 level indicated more severe, more progressive, and predicted the higher mortality and poor outcomes of ILD.

## Introduction

Interstitial lung disease (ILD) is a heterogeneous group of diseases. Despite various types of clinical presentation, the rapid progression and more severe symptoms of ILD are tending to be fatal. Within the clinical course of ILD, the severity and progression of ILD could occur at any phase closely associated with significant morbidity and mortality (1–3). Meanwhile, the global prevalence of ILD is between 10.7 and 27.14 per 100,000 people (4). In 2013, 595,000 cases a year were diagnosed with ILD worldwide and resulted in 471,000 deaths (5). However, it is not yet clear which immunological biomarker could better predict the prognosis of ILD and primarily reflect the progression and severity of ILD.

The clinical course of ILD is highly variable and unpredictable. For instance, some patients appear stable or show a slow decline of spirometry. In contrast, others show rapid deterioration, extensive lesions on high-resolution computed tomography (HRCT), or acute exacerbation of ILD (6–8). In this case, the clinician wishes for biomarkers, distinguishing between different extents or states, particularly between progressive ILD and non-progressive ILD and also between mild ILD and severe ILD.

KL-6, is a high-molecular-weight glycoprotein encoded by the MUC1 gene (Mucin 1 gene) and distributed mainly on the cell surface of type II alveolar epithelial cells (AECs) (9). When suffering from the inflammatory storm, a disulfide bond near the surface of the epithelial cell membrane of type II AECs may be disrupted, and KL-6 eventually can diffuse into the pulmonary epithelial lining fluid and blood flow (10). Therefore, KL-6 with the predictive value would predict who will be more likely to suffer from the fibrosing progressively and reflect some adverse outcomes of ILD.

This article conducts a meta-analysis and systematic review focusing on comparing groups by quantitative methods and identifying the potential risks of progression in ILD to provide the reference for clinical intervention about rapidly progressive fibrosing ILD.

## Methods

### Including Criteria

1. The quantitative data in the original study consist of the mean and standard deviation (SD) of KL-6, the number of samples in each group, or we could calculate the estimated mean (SD) through the algorithm.
2. The definitions of progressive ILD are consistent with the criteria in the INBUILD study. There are a total of four definitions about progressive ILD in clinical practice: 1) Relative decline in forced vital capacity (FVC) of ≥10% of the predictive value over 24 months despite treatment; 2) Relative decline in FVC of ≥5% to <10% of the predicted value with increased fibrosis on HRCT or with progressive symptoms over 24 months despite treatment; 3) Worsening of respiratory symptoms and increased extent of fibrosis on HRCT over 24 months despite treatment; and 4) Extent of fibrosis on HRCT >10%, FVC >45% predicted, or diffusing capacity of the lung for carbon monoxide (DLCO) ≥30% and <80% predicted.
3. Severe ILD was defined by HRCT of the chest and spirometry. ILD was considered severe when it met any of the following three criteria: 1) Severe ILD at presentation is extensive lung fibrosis on HRCT, which the extent of fibrosis on HRCT is more than 30% independently evaluated by two radiologists and two physicians with more than 18 months of experience in interstitial lung disease. 2) FVC <50%, and a requirement for continuous oxygen supplementation. 3) Oxygen partial pressure/Fraction of inspired oxygen (PaO2/FiO2) ≤300 was considered severe ILD.
4. It is mild ILD if it met any of the three criteria: 1) The extent of fibrosis on HRCT is less than 10%, independently evaluated by two radiologists and two physicians with more than 18 months of experience in interstitial lung disease. 2) FVC >50%, and with mild respiratory symptoms. 3) PaO2/FiO2 >300.5. HR and (95%CI) in the original study, which evaluates the risk factor of ILD, were adjusted. We selected the larger sample when the patients were from the same medical institution.

### Excluding Criteria

1. The content of the original study is not related to KL-6 and ILD.
2. The original study did not refer to the mean (SD) of KL-6, or we could not indirectly calculate the mean (SD) through the median (quartile).
3. HR (95%CI), in an original study evaluating the risk factor of ILD, was not adjusted.
4. Even though we contacted the corresponding author, we could not get the associated data.
5. We excluded any review, case report, animal study, and conference abstract, which could not provide the data we need.

### Data Sources and Search Strategy

**Figure 1:**
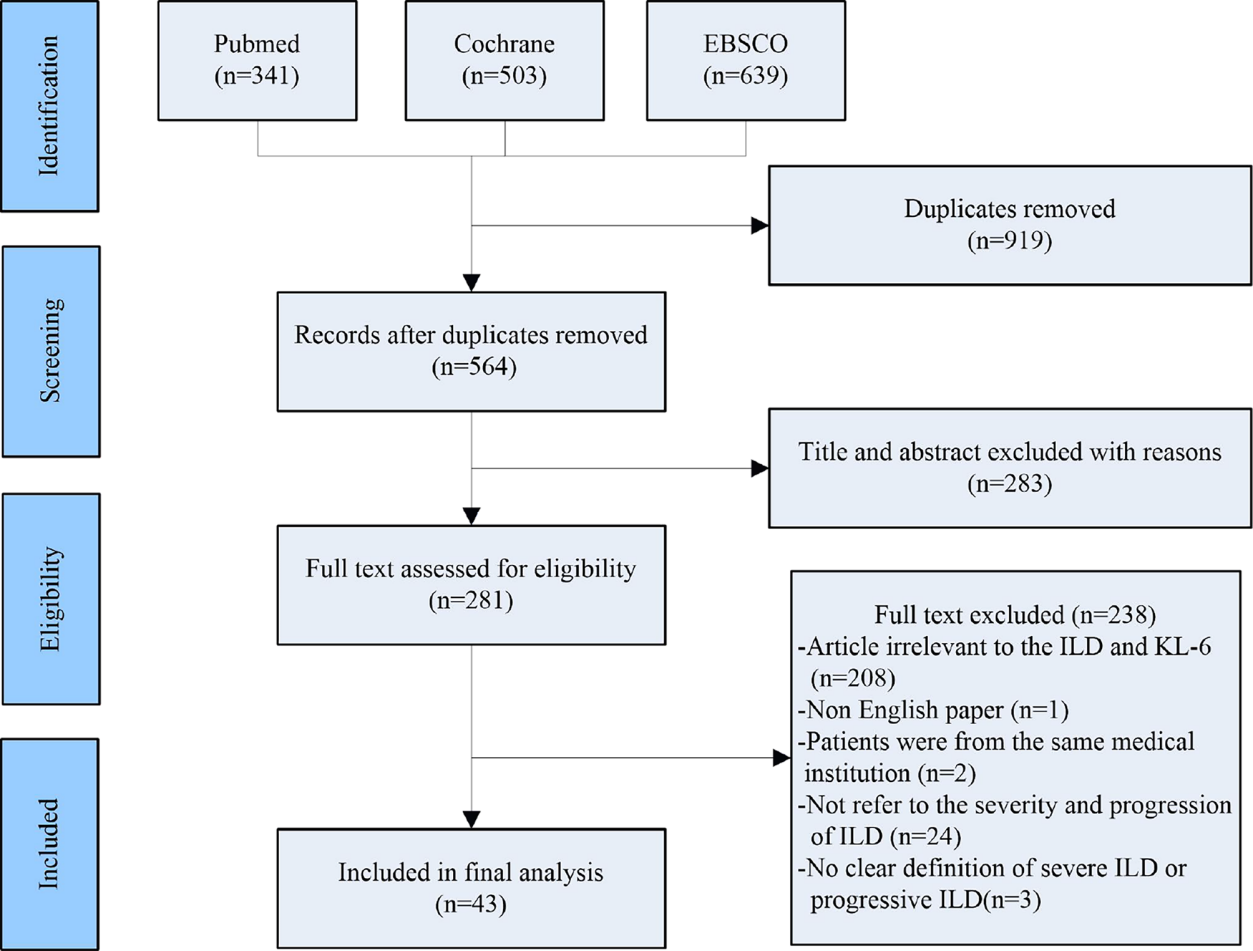
The PRISMA diagram of the study selection.

Two researchers (TZ and PS) searched the following electronic databases independently: EBSCO, PubMed and Cochrane. Search terms are as follows: ((((((progression [Title/Abstract]) OR (progressive [Title/Abstract])) AND (interstitial lung disease [Title/Abstract])) OR (ILD [Title/Abstract])) OR (ILDs [Title/Abstract])) AND (KL-6 [Title/Abstract])) OR (Krebs von den Lungen-6 [Title/Abstract]). After removing duplicates, 564 articles were screened, and the screening process of included studies are shown in **Figure 1**.

### Data Extraction and Study Selection

Firstly, two reviewers (TZ and PS) screened the titles and abstracts from the searches and independently selected relevant studies, which met the inclusion criteria. Secondly, TZ and PS respectively decided on the included and the excluded studies after reviewing the full text of these possibly included studies. Thirdly, we collected the mean (SD) of the KL-6 level and the number of samples in each group. We also collected the adjusted HR and 95%(CI), which evaluate the risk factor of adverse outcomes about ILD. Lastly, any disagreement in selection was referred to the arbitrator (LG). We also contacted the corresponding author of the original studies for additional information if our meta-analysis was needed.

### Risk of Bias (Quality) Assessment

CD and PS also independently evaluated the risk bias of included studies by the Newcastle–Ottawa Quality Assessment Scale (NOS), which mainly contains cohort studies and case–control studies. The NOS was made up of nine aspects, and each item was marked by “stars”. Marked were 7 to 9 “stars” as high-quality study; 4 to 6 “stars” were marked as moderate quality study; and 0 to 3 “stars” were marked as low-quality study. The reviewers discussed with the arbitrator (LG) when any disagreement turned up during assessment.

### Strategy for Data Synthesis

The pooled WMD 95%(CI) for comparisons between groups, the HR and 95%(CI) about elevated KL-6 level predicting adverse outcomes of ILD were calculated through a random-effects model if the I² test detected statistically significant heterogeneity (I² >50%, P <0.05), or using a fixed-effects model if otherwise. All data analyses were performed using the Meta Package in STATA, version 16.0.

## Results

### Participants/Studies to be Included

A total of 43 studies were included in our meta-analysis (11–53). The participants came from different countries, namely, China, England, Japan, Korea, Italy, and United States respectively. The participants of the original studies were mainly located in the Asian population. Among the included studies, nine studies were moderate quality studies, and 34 studies were high-quality studies.

### Search Results

Mean (SD) concentrations of KL-6 in the severe and mild groups were extracted from four studies (21, 30, 40, 42). (Mild ILD: n = 335, Severe ILD: n = 223). Mean (SD) concentrations of KL-6 for patients in progressive ILD and non-progressive ILD groups were collected from 12 studies (11, 18, 25, 28, 29, 31, 35, 36, 38, 45, 47, 53). (Progressive ILD: n = 203, Non-progressive ILD: n = 511). Mean (SD) concentrations of KL-6 for patients between a survivor of ILD and death of ILD were collected from 15 studies (12, 14, 16, 20, 23, 26, 27, 33, 34, 39, 41, 46, 48, 51, 52). (Survivor of ILD: n = 625, Death of ILD: n = 361). Meanwhile, six studies were included: mean (SD) concentrations of KL-6 both in AE-ILD and stable ILD were extracted (19, 22, 32, 43, 49, 50). (AE-ILD: n = 112, Stable ILD: n = 298). Additionally, adjusted HR (95%CI) of estimating the mortality about ILD were collected from 10 studies (13, 15, 21, 24, 26, 37, 40, 42, 48, 50), and also adjusted HR (95%CI) of estimating progression of ILD were collected from five studies (17, 21, 28, 40, 44).

### Meta-Analysis Results

**Figure 2:**
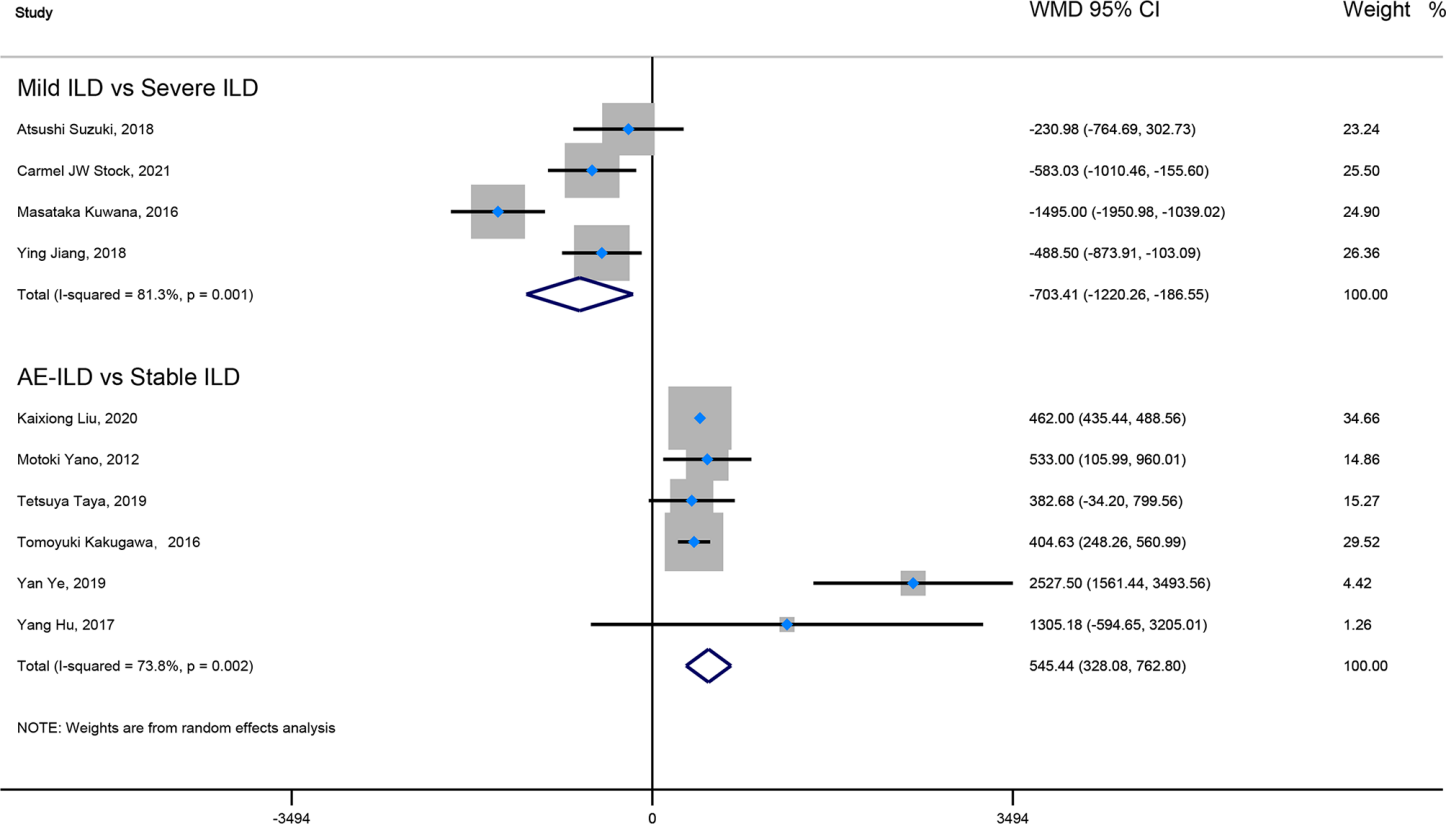
The forest plot pooled the WMD (95%CI) of KL-6 level between mild ILD and severe ILD, between AE-ILD and stable ILD.

**Figure 3:**
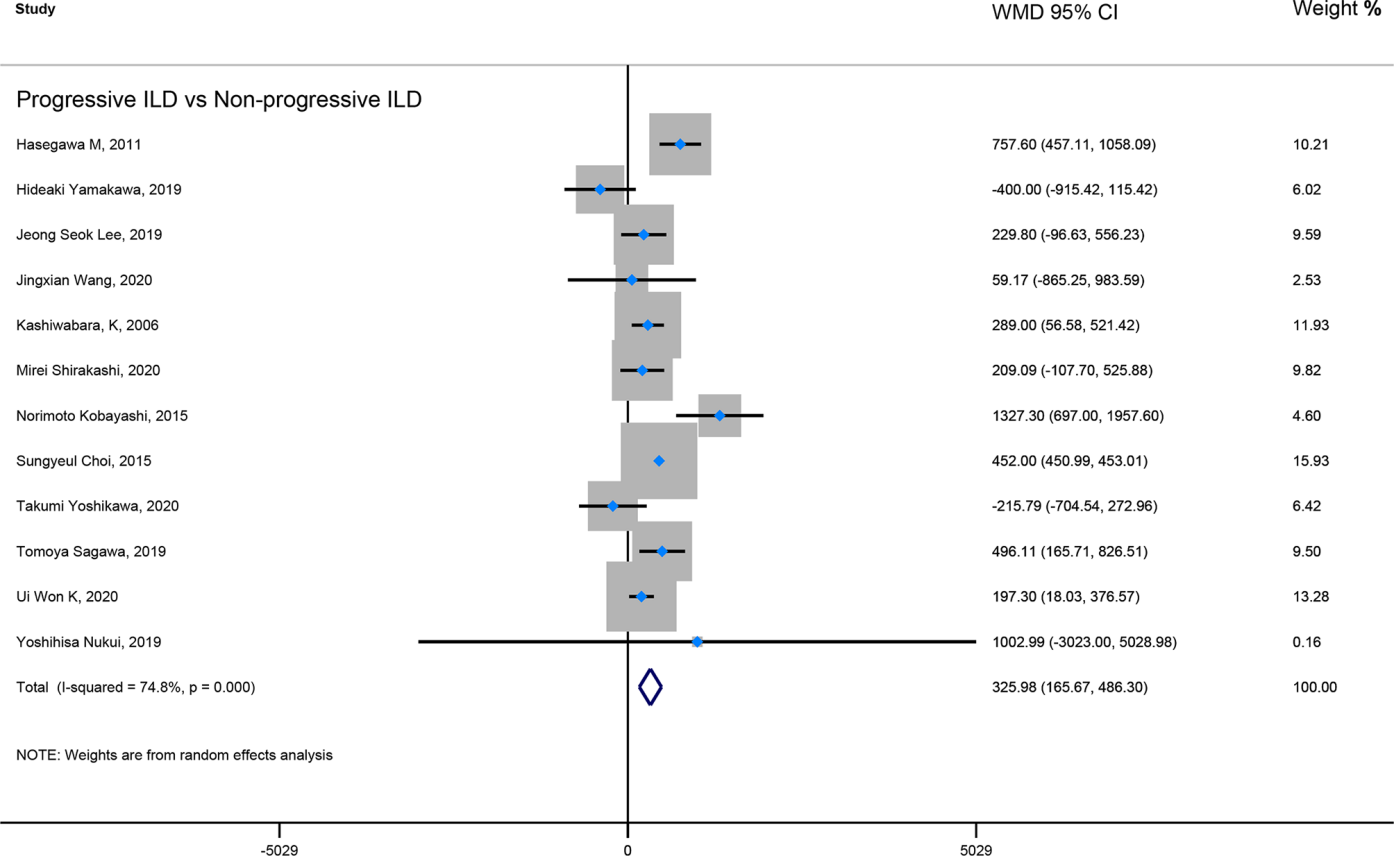
The forest plot illustrated the WMD (95%CI) of KL-6 level between progressive ILD and non-progressive ILD.

**Figure 4:**
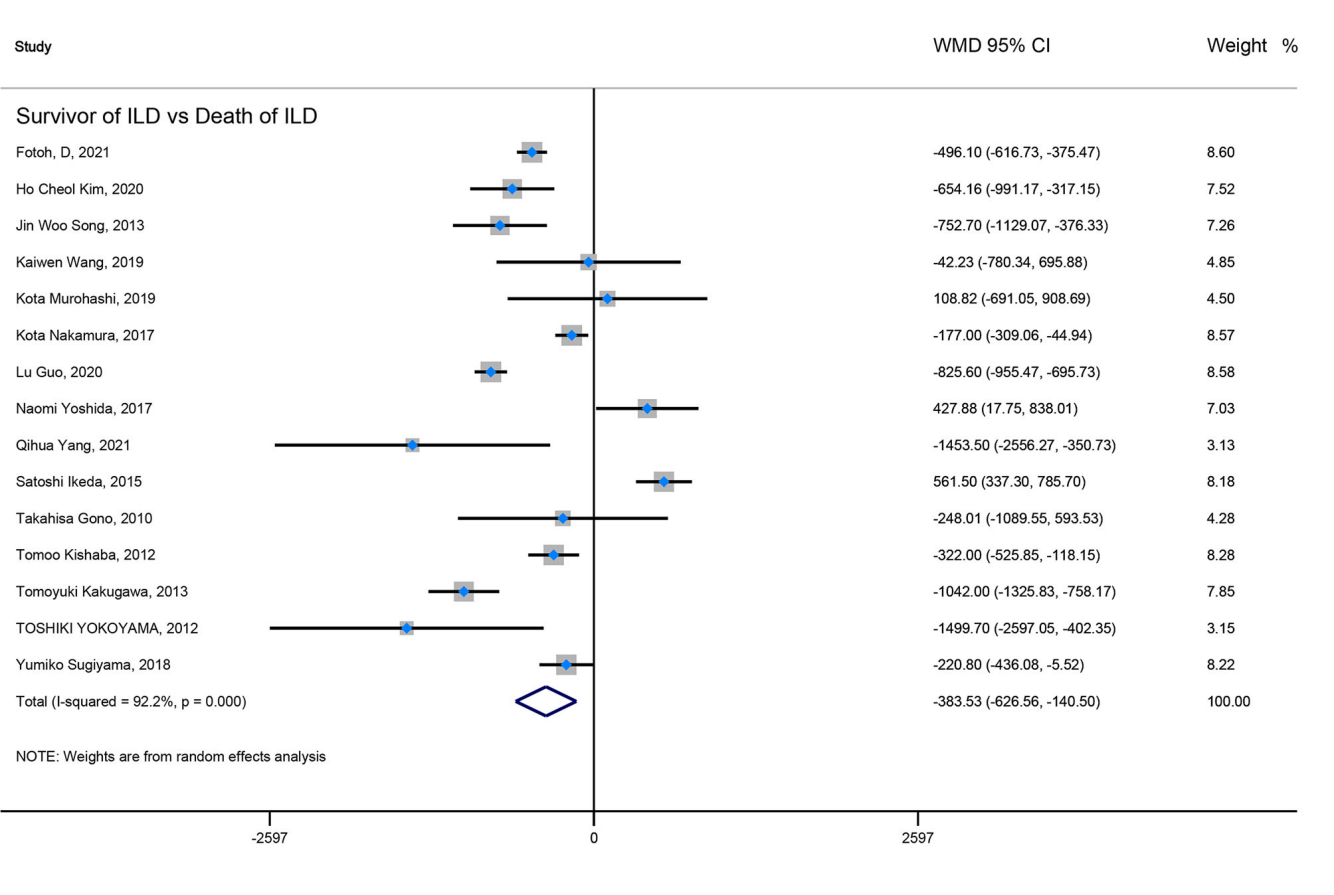
The forest plot illustrated the WMD (95%CI) of KL-6 level between ILD survivor and death of ILD.

**Figure 5:**
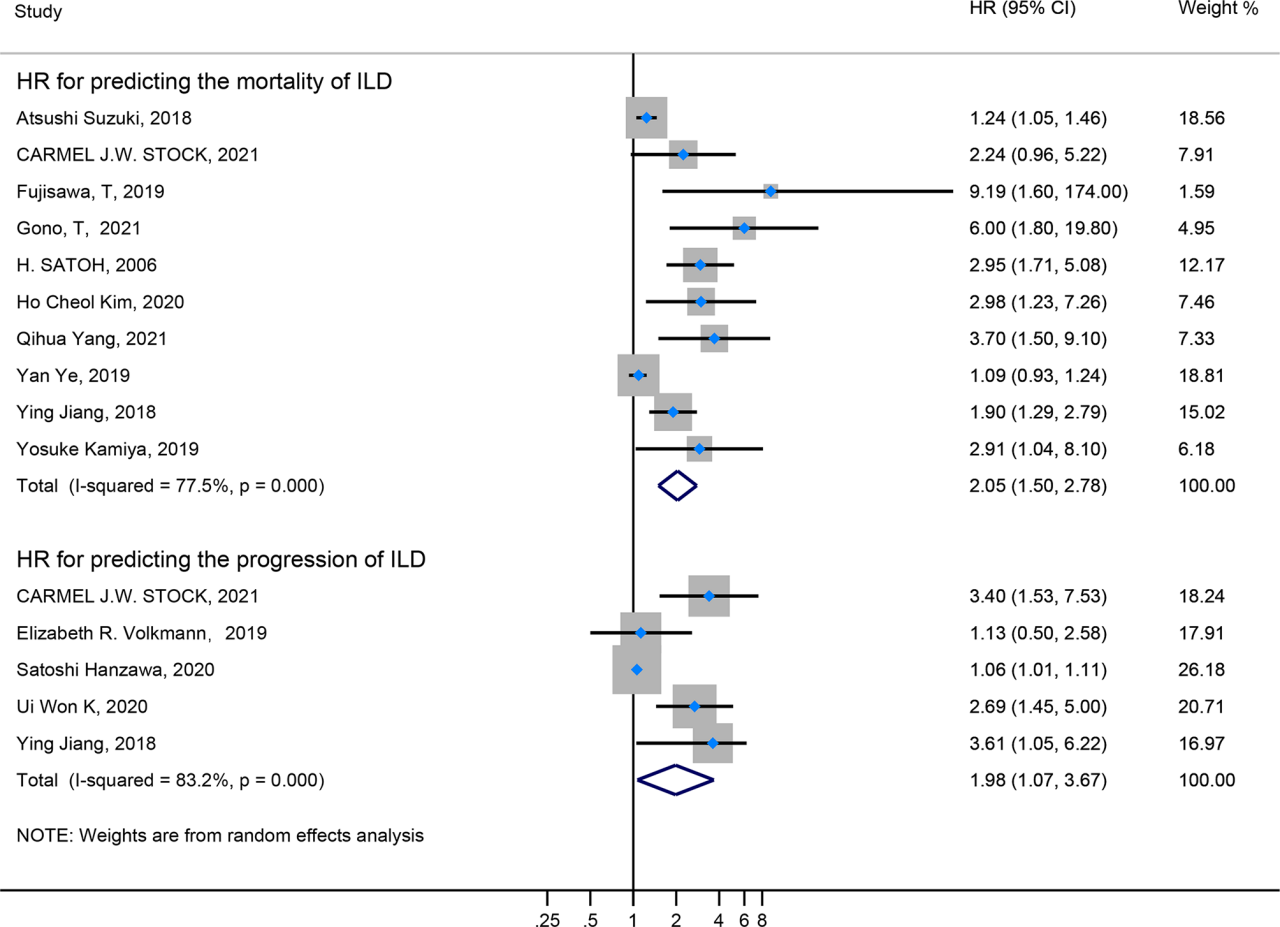
The forest plot illustrated the HR (95%CI) of elevated KL-6 level predicting the progression/mortality of ILD.

Firstly, the elevated KL-6 level will distinguish the severity and state of ILD. The KL-6 level of severe ILD was 703.41 (U/ml) higher than in mild ILD (**Figure 2**), AE-ILD was 545.44 (U/ml) higher than stable ILD (**Figure 2**). Secondly, the higher KL-6 level indicates the progression or deterioration of clinical course in ILD patients. The KL-6 level in progressive ILD was 325.98 (U/ml) higher than in non-progressive ILD (**Figure 3**), and HR (95%CI) for increased KL-6 level predicting the progression of ILD was 1.98 (1.07–3.67) (**Figure 5**), and the elevated KL-6 could effectively recognize the progressive ILD, particularly in idiopathic inflammatory myopathy associated interstitial lung disease (IIM-ILD) and indium exposure ILD (**Supplementary Figure 1**). Lastly, in terms of the survival analysis, the KL-6 level in death of ILD was 383.53 (U/ml) higher than in survivors of ILD (**Figure 4**), and highlighted that elevated KL-6 could be a prognostic marker to effectively indicate the survivor in rheumatoid arthritis-associated interstitial lung disease (RA-ILD), as well as in idiopathic pulmonary fibrosis (IPF) (**Supplementary Figure 2**). The pooled HR (95%CI) about increased KL-6 level predicting the mortality of ILD was 2.05 (1.50–2.78) (**Figure 5**).

### Sensitivity Analysis

**Figure 6:**
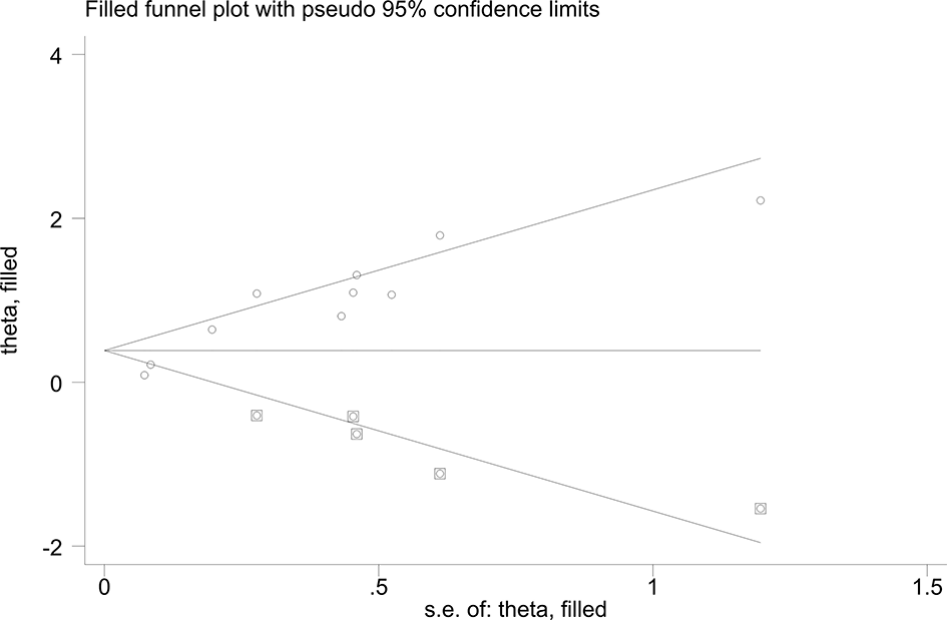
Funnel plots of nonparametric trim-and-fill method about the elevated KL-6 level predicting mortality of ILD.

In the sensitivity analysis, by excluding one study at a time, our results were stable. In terms of HR for elevated KL-6 predicting the mortality of ILD, Egger’s tests (P-value for Egger’s test = 0.000) indicated some evidence for publication bias. Next, we performed the nonparametric trim-and-fill method to evaluate the effect of possible missing studies on the overall result. We identified five studies, and the corresponding result was not significantly changed (HR = 1.238, 95%CI: 1.125–1.363), suggesting that our result was robust (**Figure 6**).

## Discussion

This study shows that elevated KL-6 takes a quantitatively distinguished effect of severity and progression for ILD and the increased KL-6 concentration predicts ILD’s poor outcomes.

Recently, some scholars rated that the persistently high levels of KL-6 were correlated with a progressively clinical course of ILD (54). Besides, an increased KL-6 level has been associated with a significant decline in FVC and DLCO (55, 56). Previous studies implicate that in some dramatically aggravated pulmonary function of ILD, whose chest HRCT demonstrated significant consolidation at the base of both lungs (57, 58). Recent studies depicted that the final diagnosis leading to death was progressive ILD accompanied by extensive ground-glass opacity (GGO) on HRCT and elevated KL-6 concentration in serum (59), and the autopsy or biopsy proved that the clinical diagnosis of progressive ILD was consistent with diffuse alveolar damage (DAD). Therefore, we may consider the consistency between the elevated KL-6 level and extensive alveolar damage of patients who are finally diagnosed with progressive ILD. In other words, the rapidly increased KL-6 level tells us to distinguish the progressive ILD from chronic ILD as early as possible. Based on previous studies, our meta-analysis concluded that patients in a progressive course of ILD have higher KL-6 levels than non-progressive patients. Still, the severe course of ILD has a higher KL-6 concentration than mild patients. The following three reasons explain why soaring the KL-6 level could effectively distinguish the severity and the different state of ILD. The first reason is that the pathological damage of ILD is varied, the most common type of which is diffuse alveolar epithelial cell injury and pulmonary vascular epithelial injury. The primary pathological mechanism of alveolar epithelial cell injury could formulate chronic fibrosis (60). Secondly, KL-6 is an immunological glycoprotein, primarily regulated by the MUC1 gene and strongly expressed on proliferated type II AECs. The increasing KL-6 concentrations usually destroy alveolar capillaries and the regeneration of type II AECs in the affected lung (61, 62). The third reason is that the severe ILD is usually accompanied by systemic inflammatory response syndrome, inflammatory exudation of degenerative type II AECs, and pulmonary consolidation on HRCT is enlarged. Therefore, more type II AECs are destroyed, and more KL-6 is released. For instance, Elhai (63) found that increased serum levels of KL -6 could be used to assess ILD severity. Even in the future, to determine whether HRCT should be performed or not in an ILD patient would be based on elevated KL-6 level as a shred of clinical evidence. Meanwhile, a study also shows that the higher serum KL-6 thresholds could aid in identifying patients who are more likely to require intensification of treatment to prevent progression of ILD (64). In this case, our pooled result concluded the critical role of elevated KL-6 level reflecting poor prognosis of ILD. In the second place, as far as the pulmonary function test, especially in the DLCO and FVC% predicted, the elevated KL-6 level also possesses the predictive value. For instance, a prospective cohort study found that when KL-6 cut-off value of 1,472 U/ml, there was a significant difference in time to decline in DLCO ≥15% (HR 3.40; 95% CI, 1.53–7.53) (40). Interestingly, another case-control research reported that an elevated serum KL-6 was with negative correlation with FVC% and forced expiratory volume in the first second (FEV1%) (r = −0.93, r = −0.91, respectively) (12). On the other hand, a recent study reported the HRCT ground-glass opacity (GGO) score ≥4 (HR 4.8; 95% CI, 1.3–17.9), KL-6 >1,600 U/ml (HR 3.7; 95% CI, 1.5–9.1) were identified as independent predictors for progressive ILD (48). To some extent, the elevated KL-6 concentrations may have consistency with the progressive lesion of HRCT under the inflammatory storm. In fact, the inflammatory pathogenic factor could initiate the basis of pulmonary fibrosis (65). Besides, an Egypt study of patients with RA-ILD demonstrated that serum KL-6 was positively correlated with HRCT scores and lung ultrasound (LUS) scores (r = 0.93, r = 0.97, respectively), and pointed increased serum KL-6 combined with LUS is an importantly new and potential prognostic factor predicting poor outcomes in RA-ILD (12).

In terms of the mortality of ILD, increased KL-6 levels could decrease the survival rate of ILD. A Chinese cohort study reported KL-6 concentration was highly elevated in progressive ILD compared with the non-progressive period (1,985.2 ± 1,497.8 vs. 1,387.6 ± 1,313.1 µg/ml), and increased KL-6 level was the best independent predictor of mortality after adjustment for other covariates (HR 1.901; 95% CI, 1.294–2.793) (21). On the other hand, a prospective study of the systemic sclerosis interstitial lung disease (SSc-ILD) demonstrated that serum KL-6 >1,273 U/ml was the most reliable predictor of end-stage lung disease development (OR 51.2; 95% CI, 7.6–343) by multivariate analysis. These patients with end-stage lung disease usually need continuous oxygen supplementation and die in a short time (30). Meanwhile, Lee et al. also indicated that those with a high KL-6 level (>933 U/ml) had a reduced survival than those without a high KL-6 level (median survival period: 51 vs. 96 months; P = 0.019) (66). Furthermore, the decreased survival rate may be due to the rapid lesion of ILD, which released a large amount of KL-6 accompanied with the severe injury of the affected lung. Indeed, some life-threatening complications such as acute respiratory distress syndrome (ARDS) and septic shock usually turn up at the stage of KL-6 level is dramatically soaring. Therefore, we may consider the internal correlation between the higher mortality and elevated KL-6 level of ILD.

Something that deserves our attention is that ILDs are a heterogeneous group of disorders, which encompass a wide range of conditions. Therefore, we classified the different types of ILD based on various etiology, such as connective tissue disease-associated interstitial lung disease (CTD-ILD), hypersensitivity pneumonia (HP), IPF, idiopathic non-specific interstitial pneumonia (iNSIP), acute exacerbation of idiopathic pulmonary fibrosis (AE-IPF), RA-ILD, IIM-ILD, and drug induced-ILD. In terms of mortality, we performed a subgroup analysis that elevated KL-6 could be a prognostic marker that can effectively recognize the survival in RA-ILD and IPF. This result could explain why KL-6 could indicate the hyperactivity of fibroblast under the diffuse alveolitis damage, and the result supports the opinion that inflammatory etiology might be the initiator of IP in ILD patients (12, 16). Meanwhile, we further found that increased KL-6 level might be an immunological biomarker to distinguish the progressive ILD, particularly in IIM-ILD and indium exposure ILD (11, 29, 36, 38). Thereby, our meta-analysis would give light to the future study, which evaluates the predictive value of KL-6 in specific ILD classification.

Note, the changes in KL-6 levels are correlated with the therapy of ILD. Previous studies pointed KL-6 level will decrease significantly after the clinical intervention compared to the pre-therapy. The two reasons for this change are explained as follows. The first reason is that both anti-fibrosis and immunosuppressive drugs could alleviate type II AECs, especially the inflammatory type II AECs (65). Therefore, the decreased inflammatory exudation will eventually result in the decline of release about the KL-6 level (67–69). The second reason is that the treatment of ILD could hinder the process of chronic fibrosis, suppress the over-proliferation of pulmonary vascular epithelial cells, and even regulate the hyperfunction of our immunological system. This therapeutic effect significantly mitigates the other lesion of ILD, so the KL-6 level will gradually drop accompanied with the suitable treatments (70, 71).

Additionally, some studies showed that the elevated KL-6 level might be a sensitive indicator for the relapse of ILD (72). We further thought about the reason for the relapse of ILD and concluded that relapse is closely related to ILD progression. The hyperactivity of pulmonary lesion on HRCT and uncontrolled destruction by systemic inflammatory response syndrome may cause the final relapse of ILD. Briefly speaking, the coincidence of increased KL-6 indicates that relapse of ILD and progression of ILD might share some common mechanism, which deserves further study to explore.

## Summary and Future Direction

This study conducts a quantitative comparison of the Kl-6 level of ILD between-groups unprecedentedly, and it also resulted in the WMD (95%CI) about KL-6 between-groups. In this case, we concluded that the elevated KL-6 level could predict the severity, progression, and poor outcomes of ILD. However, the included original studies are mainly from Asian countries. Therefore, we sincerely wish that more studies could include the non-Asian populations. Secondly, our article may imply future studies, such as the dose–response relationship between elevated KL-6 level and progression of ILD. Finally, we also expect that the future guidelines will define a definite cutoff value of KL-6. That could remind the clinical doctors to carry out the intervention of progressive ILD as early as possible.

## Data Availability Statement

The original contributions presented in the study are included in the article/**Supplementary Material**. Further inquiries can be directed to the corresponding author.

## Author Contributions

TZ and LG conceived, performed, and designed the topics. TZ gathered and read original studies, as well as wrote the first draft of the manuscript. PS and TZ extracted the data independently, which met the including criteria. TZ drew all the figures and tables. CD and PS also independently evaluated the risk bias of included studies. LG corrected and validated the manuscript. All authors contributed to the article and approved the submitted version.

## Funding

Key Projects of Sichuan Provincial Department of Science and Technology (Grant Number: 2020YFS0409) Research Fund of Sichuan Traditional Chinese Medicine Information Society (Grant Number: 201901).

## Conflict of Interest

The authors declare that the research was conducted in the absence of any commercial or financial relationships that could be construed as a potential conflict of interest.

## Publisher’s Note

All claims expressed in this article are solely those of the authors and do not necessarily represent those of their affiliated organizations, or those of the publisher, the editors and the reviewers. Any product that may be evaluated in this article, or claim that may be made by its manufacturer, is not guaranteed or endorsed by the publisher.

## Abbreviations

WMD, weighted mean difference; AE, acute exacerbation; 95%CI, 95% confidence interval; ILD, interstitial lung disease; KL-6, Krebs von den Lungen-6; HR, hazard ratio; CTD-ILD, connective tissue disease-associated interstitial lung disease; HP, hypersensitivity pneumonia; IPF, idiopathic pulmonary fibrosis; iNSIP, idiopathic non-specific interstitial pneumonia; AE-IPF, acute exacerbation of idiopathic pulmonary fibrosis; RA-ILD, rheumatoid arthritis-associated interstitial lung disease; IIM-ILD, idiopathic inflammatory myopathy associated interstitial lung disease.
