# Supplementary material for: KL-6 as an Immunological Biomarker Predicts the Severity, Progression, Acute Exacerbation, and Poor Outcomes of Interstitial Lung Disease: A Systematic Review and Meta-Analysis
Source: Front Immunol. 2021 Dec 9;12:745233. doi: 10.3389/fimmu.2021.745233 (PMC8699527; doi:10.3389/fimmu.2021.745233)
Supplement: Supplementary file 1 [file DataSheet_1.pdf]

Supplementary Material

Supplementary Table 1: Database search.

PubMed

Search	Query	Items found	Date
#1	Search progression[Title/Abstract]	563,335	2021.04.01
#2	Search progressive[Title/Abstract]	278,209	2021.04.01
#3	Search #1 OR #2	806.847	2021.04.01
#4	Search interstitial lung disease [Title/Abstract]	10,413	2021.04.01
#5	Search ILD[Title/Abstract]	4,850	2021.04.01
#6	Search ILDs[Title/Abstract]	779	2021.04.01
#7	Search #4 OR #5 OR #6	11,587	2021.04.01
#8	Search KL-6[Title/Abstract]	790	2021.04.01
#9	Search Krebs von den Lungen-6 [Title/Abstract]	259	2021.04.01
#10	Search #8 OR #9	856	2021.04.01
#11	Search #3 AND #7 AND #10	341	2021.04.01

EBSCO

Search	Query	Items found	Date
#1	Search progression:ab,ti	15,985	2021.04.01
#2	Search progressive:ab,ti	24,448	2021.04.01
#3	Search #1 OR #2	40,383	2021.04.01
#4	Search interstitial lung disease:ab,ti	1997	2021.04.01
#5	Search ILD:ab,ti	1108	2021.04.01
#6	Search ILDs:ab,ti	1108	2021.04.01
#7	Search #4 OR #5 OR #6	1750	2021.04.01
#8	Search KL-6;ab,ti	170	2021.04.01
#9	Search Krebs von den Lungen-6:ab,ti	6	2021.04.01
#10	Search #8 OR #9	116	2021.04.01
#11	Search #3 AND #7 AND #10	639	2021.04.01

Cochrane

Search		Items found	Date
#1	Search (progression):ti,ab,kw	98,990	2021.04.01
#2	Search (progressive):ti,ab,kw	98,972	2021.04.01
#3	Search #1 OR #2	98,993	2021.04.01
#4	Search (interstitial lung disease):ti,ab,kw	1,507	2021.04.01
#5	Search (ILD):ti,ab,kw	606	2021.04.01
#6	Search (ILDs):ti,ab,kw	594	2021.04.01
#7	Search #4 OR #5 #6	1,614	2021.04.01
#8	Search (KL-6):ti,ab,kw	65	2021.04.01
#9	Search (Krebs von den Lungen-6):ti,ab,kw	17	2021.04.01
#10	Search #8 OR #9	72	2021.04.01
#11	Search #3 AND #7 AND #10	503	2021.04.01

Supplementary Table 2: Reasons for study exclusion.

(1) No available data about KL-6 and ILD or not refer to the severity and progression of ILD (N=24).

1.Al-Salmi, Q. A., Walter, J. N., Colasurdo, G. N., Sockrider, M. M., Smith, E. O., Takahashi, H., & Fan, L. L. (2005). Serum KL-6 and surfactant proteins A and D in pediatric interstitial lung disease. *Chest, 127*(1), 403-407. doi:10.1378/chest.127.1.403

2.Benyamine, A., Heim, X., Resseguier, N., Bertin, D., Gomez, C., Ebbo, M., . . . Granel, B. (2018). Elevated serum Krebs von den Lungen-6 in systemic sclerosis: a marker of lung fibrosis and severity of the disease. *Rheumatol Int, 38*(5), 813-819. doi:10.1007/s00296-018-3987-3

3.Bergantini, L., d'Alessandro, M., Vietri, L., Rana, G. D., Cameli, P., Acerra, S., . . . Bargagli, E. (2020). Utility of serological biomarker' panels for diagnostic accuracy of interstitial lung diseases. *Immunol Res, 68*(6), 414-421. doi:10.1007/s12026-020-09158-0

4.Cummings, K. J., Virji, M. A., Trapnell, B. C., Carey, B., Healey, T., & Kreiss, K. (2014). Early changes in clinical, functional, and laboratory biomarkers in workers at risk of indium lung disease. *Ann Am Thorac Soc, 11*(9), 1395-1403. doi:10.1513/AnnalsATS.201407-346OC

5.d'Alessandro, M., Bergantini, L., Cameli, P., Pieroni, M., Refini, R. M., Sestini, P., & Bargagli, E. (2021). Serum Concentrations of KL-6 in Patients with IPF and Lung Cancer and Serial Measurements of KL-6 in IPF Patients Treated with Antifibrotic Therapy. *Cancers (Basel), 13*(4). doi:10.3390/cancers13040689

6.Endo, Y., Koga, T., Suzuki, T., Hara, K., Ishida, M., Fujita, Y., . . . Kawakami, A. (2018). Successful treatment of plasma exchange for rapidly progressive interstitial lung disease with anti-MDA5 antibody-positive dermatomyositis: A case report. *Medicine (Baltimore), 97*(15), e0436. doi:10.1097/md.0000000000010436

7.Fathi, M., Barbasso Helmers, S., & Lundberg, I. E. (2012). KL-6: a serological biomarker for interstitial lung disease in patients with polymyositis and dermatomyositis. *J Intern Med, 271*(6), 589-597. doi:10.1111/j.1365-2796.2011.02459.x

8.Frix, A. N., Schoneveld, L., Ladang, A., Henket, M., Duysinx, B., Vaillant, F., . . . Guiot, J. (2020). Could KL-6 levels in COVID-19 help to predict lung disease? *Respir Res, 21*(1), 309. doi:10.1186/s12931-020-01560-4

9.Furukawa, H., Oka, S., Shimada, K., Hashimoto, A., Komiya, A., Matsui, T., & Tohma, S. (2019). Role of Deleterious Rare Alleles for Acute-Onset Diffuse Interstitial Lung Disease in Collagen Diseases. *Clin Med Insights Circ Respir Pulm Med, 13*, 1179548419866443. doi:10.1177/1179548419866443

10.Hamada, T., Samukawa, T., Kumamoto, T., Hatanaka, K., Tsukuya, G., Yamamoto, M., . . . Inoue, H. (2015). Serum B cell-activating factor (BAFF) level in connective tissue disease associated interstitial lung disease. *BMC Pulm Med, 15*, 110. doi:10.1186/s12890-015-0105-0

11.Hua-Huy, T., Rivière, S., Tiev, K. P., & Dinh-Xuan, A. T. (2014). [Use of pulmonary function tests and biomarkers studies to diagnose and follow-up interstitial lung disease in systemic sclerosis]. *Rev Pneumol Clin, 70*(6), 335-342. doi:10.1016/j.pneumo.2014.09.003

12.Ikuyama, Y., Ushiki, A., Kosaka, M., Akahane, J., Mukai, Y., Araki, T., . . . Hanaoka, M. (2020). Prognosis of patients with acute exacerbation of combined pulmonary fibrosis and emphysema: a retrospective single-centre study. *BMC Pulm Med, 20*(1), 144. doi:10.1186/s12890-020-01185-9

13.Inoue, Y., Kaner, R. J., Guiot, J., Maher, T. M., Tomassetti, S., Moiseev, S., . . . Brown, K. K. (2020). Diagnostic and Prognostic Biomarkers for Chronic Fibrosing Interstitial Lung Diseases With a Progressive Phenotype. *Chest, 158*(2), 646-659. doi:10.1016/j.chest.2020.03.037

14.Ishiguro, T., Yasui, M., Takato, H., Kimura, H., Katayama, N., Kasahara, K., & Fujimura, M. (2007). Progression of interstitial lung disease upon overlapping of systemic sclerosis with polymyositis. *Intern Med, 46*(15), 1237-1241. doi:10.2169/internalmedicine.46.0135

15.Ishii, H., Kinoshita, Y., Kushima, H., Nagata, N., & Watanabe, K. (2019). The similarities and differences between pleuroparenchymal fibroelastosis and idiopathic pulmonary fibrosis. *Chron Respir Dis, 16*, 1479973119867945. doi:10.1177/1479973119867945

16.Kamio, K., Azuma, A., Ohta, K., Sugiyama, Y., Nukiwa, T., Kudoh, S., & Mizushima, T. (2014). Double-blind controlled trial of lecithinized superoxide dismutase in patients with idiopathic interstitial pneumonia - short term evaluation of safety and tolerability. *BMC Pulm Med, 14*, 86. doi:10.1186/1471-2466-14-86

17.Kaneko, Y., Nunokawa, T., Taniguchi, Y., Yamaguchi, Y., Gono, T., Masui, K., . . . Kuwana, M. (2020). Clinical characteristics of cancer-associated myositis complicated by interstitial lung disease: a large-scale multicentre cohort study. *Rheumatology (Oxford), 59*(1), 112-119. doi:10.1093/rheumatology/kez238

18.Kawase, S., Hattori, N., Ishikawa, N., Horimasu, Y., Fujitaka, K., Furonaka, O., . . . Kohno, N. (2011). Change in serum KL-6 level from baseline is useful for predicting life-threatening EGFR-TKIs induced interstitial lung disease. *Respir Res, 12*(1), 97. doi:10.1186/1465-9921-12-97

19.Mimori, T., Nakashima, R., & Hosono, Y. (2012). Interstitial lung disease in myositis: clinical subsets, biomarkers, and treatment. *Curr Rheumatol Rep, 14*(3), 264-274. doi:10.1007/s11926-012-0246-6

20.Miyazaki, K., Kurishima, K., Kagohashi, K., Kawaguchi, M., Ishikawa, H., Satoh, H., & Hizawa, N. (2010). Serum KL-6 levels in lung cancer patients with or without interstitial lung disease. *J Clin Lab Anal, 24*(5), 295-299. doi:10.1002/jcla.20404

21.Mizuno, R., Asano, K., Mikami, S., Nagata, H., Kaneko, G., & Oya, M. (2012). Patterns of interstitial lung disease during everolimus treatment in patients with metastatic renal cell carcinoma. *Jpn J Clin Oncol, 42*(5), 442-446. doi:10.1093/jjco/hys033

22.Okuda, R., Matsushima, H., Aoshiba, K., Oba, T., Kawabe, R., Honda, K., & Amano, M. (2015). Soluble intercellular adhesion molecule-1 for stable and acute phases of idiopathic pulmonary fibrosis. *Springerplus, 4*, 657. doi:10.1186/s40064-015-1455-z

23.Salazar, G. A., Kuwana, M., Wu, M., Estrada, Y. M. R. M., Ying, J., Charles, J., . . . Assassi, S. (2018). KL-6 But Not CCL-18 Is a Predictor of Early Progression in Systemic Sclerosis-related Interstitial Lung Disease. *J Rheumatol, 45*(8), 1153-1158. doi:10.3899/jrheum.170518

24.Shimoda, M., Tanaka, Y., Fujiwara, K., Furuuchi, K., Osawa, T., Morimoto, K., . . . Ohta, K. (2021). Waterproofing spray-associated pneumonitis review: Comparison with acute eosinophilic pneumonia and hypersensitivity pneumonitis. *Medicine (Baltimore), 100*(10), e25054. doi:10.1097/md.0000000000025054

(2) No clear definition of severe ILD or progressive ILD(N=3)

1.Cao, X. , Hu, S. , Xu, D. , Li, M. , Wang, Q. , & Hou, Y. , et al. (2019). Serum levels of krebs von den lungen - 6 as a promising marker for predicting occurrence and deterioration of systemic sclerosis - associated interstitial lung disease from a chinese cohort. Wiley-Blackwell Online Open , 22(1). doi： 10.1111/1756-185X.13452

2.Arai, T. , Akira, M. , Sugimoto, C. , Tachibana, K. , & Inoue, Y. . (2020). Seroradiologic prognostic evaluation of acute exacerbation in patients with idiopathic interstitial pneumonia: a retrospective observational study. Journal of Thoracic Disease, 12(8), 4132-4147. doi： 10.21037/jtd-20-911

3. Kobayashi, N. , Kobayashi, I. , Mori, M. , Sato, S. , Iwata, N. , & Shigemura, T. , et al. (2015). Increased serum b cell activating factor and a proliferation-inducing ligand are associated with interstitial lung disease in patients with juvenile dermatomyositis. Journal of Rheumatology, 2412-8. doi:10.3899/jrheum.140977

(3) Non-English article（N=1）

1. Hua-Huy, T. , S Rivière, Tiev, K. P. , & Dinh-Xuan, A. T. . (2014). [use of pulmonary function tests and biomarkers studies to diagnose and follow-up interstitial lung disease in systemic sclerosis.]. Revue de Pneumologie Clinique, 70(6), 335-42. doi：10.1016/j.pneumo.2014.09.003

(4) Patients were from the same medical institution(N=2)

1. Choi, S. , Won, Y. L. , & D Kim…. (2013). Subclinical interstitial lung damage in workers exposed to indium compounds. Annals of Occupational & Environmental Medicine, 25(1), 24-24. doi： 10.1186/2052-4374-25-24

2. Kakugawa, T. , Yokota, S. I. , Ishimatsu, Y. , Hayashi, T. , Nakashima, S. , & Hara, S. , et al. (2014). Serum heat shock protein 47 levels are elevated in acute interstitial pneumonia. BMC Pulmonary Medicine,14,1(2014-03-21), 14. doi： 10.1186/1471-2466-14-48

Supplementary Table 3: The included studies depicted the difference of KL-6 level between mild ILD and severe ILD.

author	study	country	follow up	KL-6 in Mild ILD (U/ml)			KL-6 in Severe ILD (U/ml)		
				Mean	SD	Sample	Mean	SD	Sample
Atsushi Suzuki, 2018	cohort study	Japan	0.25	1811.75	1138.07	49	2042.73	787.62	13
Carmel JW Stock, 2021	cohort study	UK	4	1234.11	974.35	42	1817.14	1340.45	72
Masataka Kuwana, 2016	cohort study	Japan	14.45	694	322	34	2189	904	16
Ying Jiang, 2018	observational study	China	1	996	1343.3	210	1484.5	1915.5	122

Supplementary Table 4: The included studies depicted the difference of KL-6 level between progressive ILD and non-progressive ILD.

author	study	country	follow up	KL-6 in Progressive ILD (U/ml)			KL-6 in Non-progressive ILD (U/ml)		
				Mean	SD	Sample	Mean	SD	Sample
Hasegawa M, 2011	longitudinal study	Japan	NG	1225.1	630.7	18	467.5	231.2	38
Hideaki Yamakawa, 2019	retrospective study	Japan	12.5	1456	730.6	13	1856	1319.8	62
Jeong Seok Lee, 2019	retrospective study	Korean	13	957.2	720.2	28	727.4	773.9	65
Jingxian Wang, 2020	retrospective study	China	0.25	1149.23	1523.82	13	1090.06	783.39	14
Kashiwabara, K, 2006	observational study	Japan	NG	607	297	7	318	143	14
Mirei Shirakashi, 2020	retrospective study	Japan	0.5	806.47	559.31	13	597.38	226.96	25
Norimoto Kobayashi, 2015	case control study	Japan	4	2045.6	881.5	10	718.3	699	19
Sungyeul Choi, 2015	observational study	Korean	1.4	1179.9	1.5	12	727.9	1.7	38
Takumi Yoshikawa, 2020	cohort study	Japan	0.5	1069.57	537.01	17	1285.36	1202.95	32
Tomoya Sagawa, 2019	cohort study	Japan	8.8	1191.26	525.01	15	695.15	374.93	14
Ui Won K, 2020	cohort study	Korean	0.925	826.3	531.6	43	629	529	156
Yoshihisa Nukui, 2019	retrospective study	Japan	0.5	2440.29	7654.62	14	1437.3	1077.72	34

Supplementary Table 5: The included studies depicted the difference of KL-6 level between survivors of ILD and death of ILD.

author	study	country	follow up	KL-6 in Survivor of ILD(U/ml)			KL-6 in Death of ILD (U/ml)		
				Mean	SD	Sample	Mean	SD	Sample
Fotoh, D, 2021	case-control study	America	1	860.1	424.1	50	1356.2	69.1	25
Ho Cheol Kim, 2020	retrospective study	Korea	5.08	699.5	403.32	51	1353.66	932.95	33
Jin Woo Song, 2013	retrospective study	Korea	2	1099	796.1	54	1851.7	1268.4	64
Kaiwen Wang, 2019	case control study	China	0.5	1234.54	1694.12	68	1276.77	1301.34	17
Kota Murohashi, 2019	retrospective study	Japan	0.25	1492.07	1675.32	48	1383.25	1470.21	20
Kota Nakamura, 2017	case control study	Japan	0.125	137	147.62	24	314	208.77	12
Lu Guo, 2020	cohort study	China	5	535.3	214.2	57	1360.9	497.4	69
Naomi Yoshida, 2017	case control study	Japan	0.25	1171.81	823.48	18	743.93	156.39	4
Qihua Yang, 2021	cohort study	China	0.5	973.27	488.35	68	2426.77	2624.4	22
Satoshi Ikeda, 2015	retrospective study	Japan	NG	1097.5	212.5	4	536	103.8	6
Tomoo Kishaba, 2012	cohort study	Japan	5	852	278	26	1174	725	67
Takahisa Gono, 2010	retrospective study	Japan	17	809.44	611.86	9	1057.45	844.86	5
Tomoyuki Kakugawa, 2013	retrospective study	Japan	NG	502	935	44	1544	57.5	3
TOSHIKI YOKOYAMA, 2012	cohort study	Japan	0.274	1515.7	837.3	26	3015.4	1854.2	12
Yumiko Sugiyama, 2018	case control study	Japan	0.5	807.22	614.47	102	1028.02	342.16	14

Supplementary Table 6: The included studies depicted the difference of KL-6 level between AE-ILD and stable ILD.

author	study	country	follow up	KL-6 in AE of ILD (U/ml)			KL-6 in stable ILD (U/ml)		
				Mean	SD	Sample	Mean	SD	Sample
Kaixiong Liu, 2020	case-control study	China	2	1958	61.51	34	1496	59.57	49
Motoki Yano, 2012	cohort study	Japan	6.66	1143	518	6	610	392	56
Tetsuya Taya, 2019	retrospective study	Japan	4	1011.69	768.72	14	629.01	504.52	84
Tomoyuki Kakugawa，2016	cohort study	Japan	2.6	1316.625	315.375	24	912	301.75	41
Yan Ye, 2019	cohort study	China	1	3262	2124	19	734.5	331.8	20
Yang Hu, 2017	case control study	China	NG	2351.53	3697.22	15	1046.35	1165.09	48

Supplementary Table 7: The included studies depicted the high KL-6 level predicting the hazard ratio of progression about ILD.

author	study	country	HR	Low limitation	Upper limitation	P value
CARMEL J.W. STOCK, 2021	cohort study	UK	3.4	1.53	7.53	0.003
Elizabeth R. Volkmann，2019	cohort study	Italy	1.13	0.5	2.58	0.0767
Satoshi Hanzawa, 2020	observational study	Japan	1.06	1.01	1.11	0.01
Ui Won K, 2020	cohort study	Korea	2.689	1.445	5.004	0.002
Ying Jiang, 2018	observational study	China	3.61	1.05	6.22	0.01

Supplementary Table 8: The included studies depicted the high KL-6 level predicting the hazard ratio of mortality about ILD.

author	study	country	HR	Low limitation	Upper limitation	P value
Atsushi Suzuki, 2018	cohort study	Japan	1.239	1.05	1.461	0.011
CARMEL J.W. STOCK, 2021	cohort study	UK	2.24	0.96	5.22	0.06
Fujisawa, T, 2019	retrospective study	Japan	9.19	1.6	174	0.01
Gono, T, 2021	cohort study	Japan	6.0	1.8	19.8	0.003
H. SATOH, 2006	prospective study	Japan	2.95	1.71	5.08	0.0001
Ho Cheol Kim, 2020	retrospective study	Korea	2.984	1.227	7.257	0.016
Qihua Yang, 2021	cohort study	China	3.7	1.5	9.1	0.004
Yan Ye, 2019	cohort study	China	1.09	0.932	1.241	0.041
Ying Jiang, 2018	observational study	China	1.901	1.294	2.793	0.001
Yosuke Kamiya, 2019	observational study	Japan	2.91	1.04	8.10	0.04

ILD: Interstitial Lung Disease
KL-6: Krebs von den Lungen-6
AE: Acute Exacerbation
HR: Hazard Ratio

Supplementary Figure 1: The subgroup analysis in various specific ILDs about the WMD (95%CI) of KL-6 level between progressive ILD and non-progressive ILD.

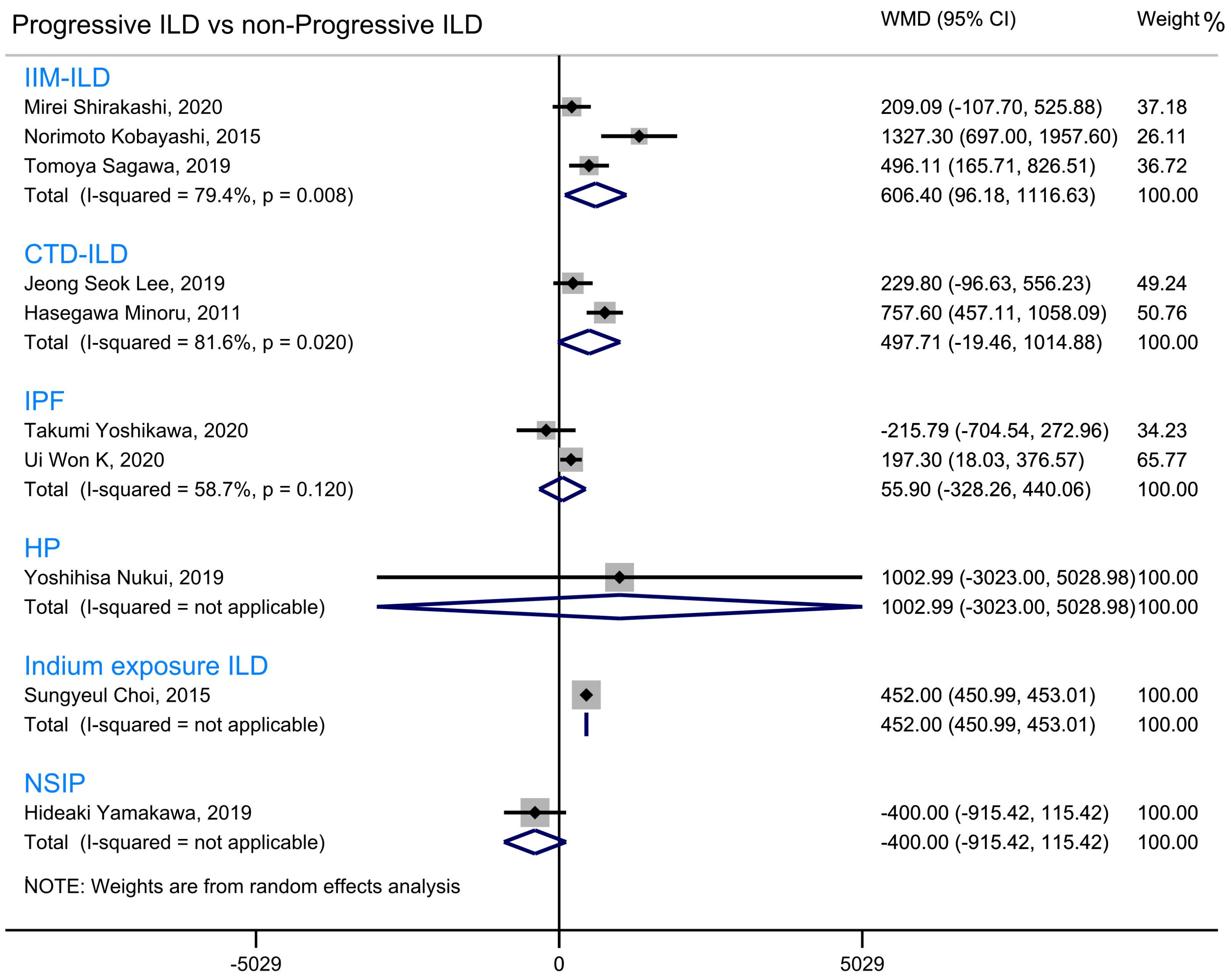

IIM-ILD: idiopathic inflammatory myopathy associated interstitial lung disease

CTD-ILD: connective tissue disease-associated interstitial lung disease

IPF: idiopathic pulmonary fibrosis

HP: hypersensitivity pneumonia

Drug induced ILD: Drug induced interstitial lung disease

NSIP: idiopathic non-specific interstitial pneumonia

Supplementary Figure 2: The subgroup analysis in various specific ILDs about the WMD (95%CI) of KL-6 level between ILD survivor and death of ILD.

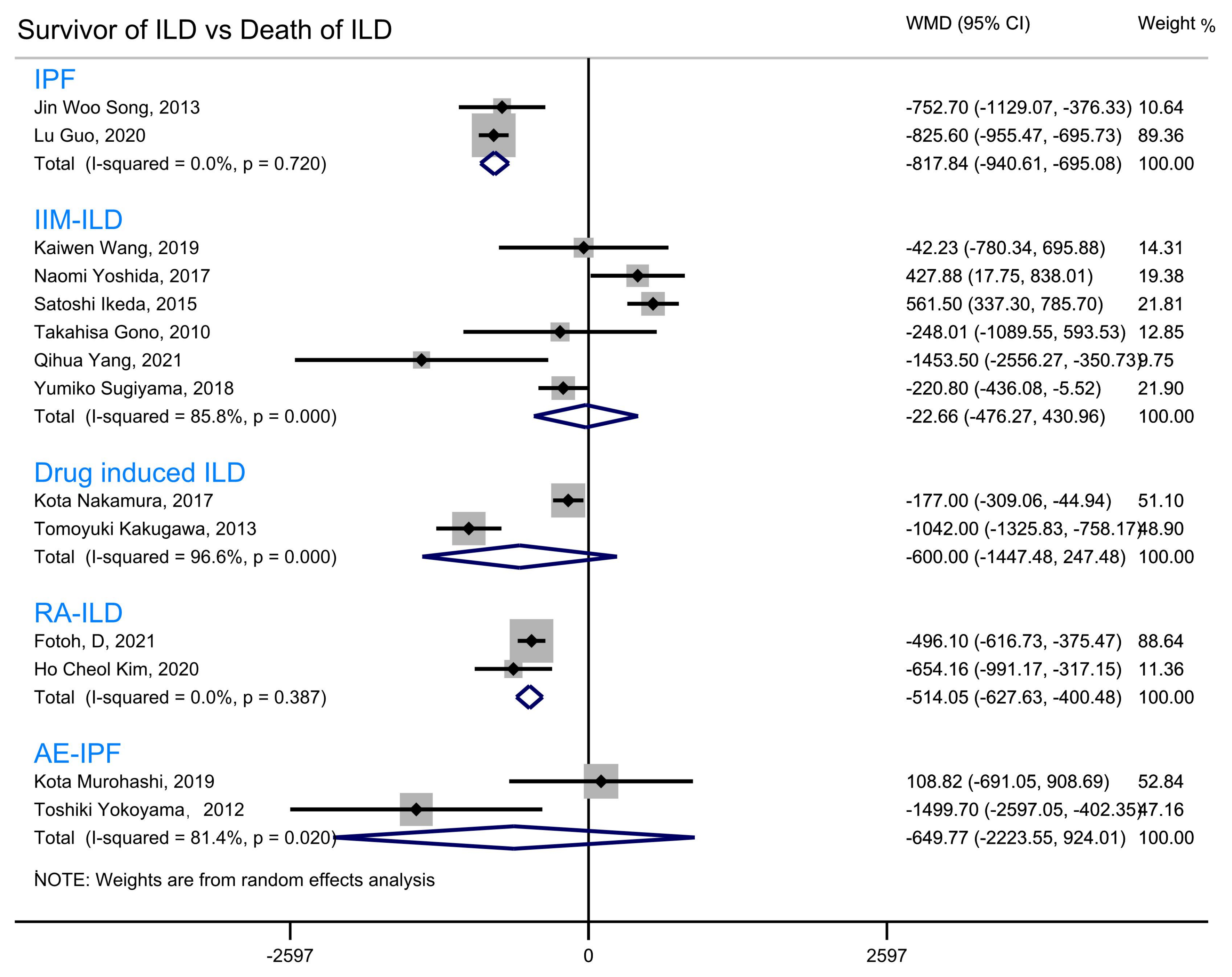

IPF: idiopathic pulmonary fibrosis

IIM-ILD: idiopathic inflammatory myopathy associated interstitial lung disease

Drug induced ILD: Drug induced interstitial lung disease

RA-ILD: rheumatoid arthritis-associated interstitial lung disease

AE-IPF: acute exacerbation of idiopathic pulmonary fibrosis
